# The mitochondrial genome of the land snail *Camaena cicatricosa* (Müller, 1774) (Stylommatophora, Camaenidae): the first complete sequence in the family Camaenidae

**DOI:** 10.3897/zookeys.451.8537

**Published:** 2014-11-03

**Authors:** Pei Wang, Hai-Fan Yang, Wei-Chuan Zhou, Chung-Chi Hwang, Wei-Hong Zhang, Zhou-Xing Qian

**Affiliations:** 1 Key Laboratory of Molluscan Quarantine and Identification of AQSIQ, Fujian Entry-Exit Inspection & Quarantine Bureau, Fuzhou, Fujian 350001, China; 2 National Wetland Museum of China, Hangzhou, Zhejiang 310013, China; 3 Department of Life Sciences, National University of Kaohsiung, No.700, Kaohsiung University Road, Nan-Tzu District, Kaohsiung 81148, Taiwan; 4 College of Life Science and Technology, Xinjiang University, Urumqi, Xinjiang 830046, China; 5 Zhejiang Museum of Natural History, Hangzhou, Zhejiang 310014, China

**Keywords:** *Camaena cicatricosa*, Camaenidae, Stylommatophora, mitochondrial genome, secondary structure

## Abstract

The complete mitochondrial (mt) genome of the snail *Camaena cicatricosa* (Müller, 1774) has been sequenced and annotated in this study. The entire circular genome is 13,843 bp in size and represents the first camaenid mt genome, with content of 31.9%A, 37.9%T, 13.5%C and 16.7%G. Gene content, codon usage and base organization show similarity to a great extent to the sequenced mt genome from Stylommatophora, whereas, gene order is different from them, especially the positions of *tRNA^Cys^*, *tRNA^Phe^*, *COII*, *tRNA^Asp^*, *tRNA^Gly^*, *tRNA^His^* and *tRNA^Trp^*. All protein coding genes use standard initiation codons ATN except for *COII* with GTG as start signal. Conventional stop codons TAA and TAG have been assigned to all protein coding genes. All tRNA genes possess the typical clover leaf structure, but the TψC arm of *tRNA^Asp^* and dihydrouridine arm of *tRNA^Ser(AGN)^* only form a simple loop. Shorter intergenic spacers have been found in this mt genome. Phylogenetic study based on protein coding genes shows close relationship of Camaenidae and Bradybaenidae. The presented phylogeny is consistent with the monophyly of Stylommatophora.

## Introduction

The mitochondrial (mt) genome of metazoa usually comprise 37 genes, including 13 protein coding genes (PCGs) (*COI*−*COIII*, *Cytb*, *ND1*−*ND6*, *ND4L*, *ATP6* and *ATP8*), two ribosomal RNA (rRNA) genes, and 22 transfer RNA (tRNA) genes (Boore 1999). Additionally, it also contains noncoding regions, such as the AT-rich region and short intergenic spacers (Wolstenholme 1992). The mt genome is characterized by small size (13−36 kb), maternal inheritance, lack of recombination, conserved genomic organization and rapid evolutionary rate compared with the nuclear genome (Avise 1994). It has been widely used in studies of systematics, phylogenetic analysis, phylogeography, population structure at diverse taxonomic groups (White et al. 2011; Gaitán-Espitia et al. 2013; Menegon et al. 2014). The mt genome is the most popular genetic marker though there are numerous debates on their utilization in systematic research (Delsuc et al. 2003; Cameron et al. 2004; Talavera and Vila 2011; Simon and Hadrys 2013; Cameron 2014). Over the last years, next generation sequencing technologies have accelerated further developments of mt genomics. The mt genomes of many vertebrates and insects are well sequenced and studied (Boore 1999; Hahn et al. 2013; Wang et al. 2014). However, studies on molluscan mt genomes are poor relatively (Kurabayashi and Ueshima 2000; Boore et al. 2004; Grande et al. 2008). Only 80 mt genomes of Gastropoda snails have been deposited in GenBank (up to 2014.9.20).

Camaenidae, one of the most diverse families, was erected by Pilsbry in 1893 (Pilsbry 1893–1895). The camaenids mainly feed on green plants and humus, and often harm a large number of crops, landscape plants and forest, leading to a depression in yield and a reduction in quality. Besides, they can spread zoonotic food borne parasitic disease and have great damage to human and animal health (Zhou et al. 2007). When humans are infected by ingesting snails, the nervous system can be injured (Liang and Pan 1992). The camaenids also play an important part in agricultural production and human activities as food, drug, arts, crafts, etc. (Chen and Gao 1987). *Camaena cicatricosa* (Müller, 1774), the type species of the type genus *Camaena* (Albers, 1850), occurs only in China, distributing in Guangdong, Guangxi, Guizhou, Yunnan and Hainan. Adult shell is large, thick and depressed conic. This snail usually feeds a broad range of fruits, vegetables, leaves and weeds (Xiao 1989).

The mt genome of land snail is similar to other invertebrates in containing 37 genes. Since the first mt genome of *Albinaria caerulea* was obtained in 1995 (Hatzoglou et al. 1995), only ten mt genomes from eight species in the order Stylommatophora were determined prior to this study, consisting of three species in Helicidae (Terrett et al. 1995; Groenenberg et al. 2012; Gaitán-Espitia et al. 2013), two in Bradybaenidae (Yamazaki et al. 1997; Deng et al. 2014), one in Clausiliidae (Hatzoglou et al. 1995), one in Succineidae (White et al. 2011) and one in Achatinidae (He et al. 2014). Although researchers have done some phylogenetic studies on Camaenidae, they often pay much attention to analyses of shell morphology or single gene fragment (Scott 1996; Wade et al. 2007). Complete mt genome evidence is still limited. We select *Camaena cicatricosa* as subject because of not only relatively wide distribution and varied morphology but also acting as type species of the type genus *Camaena*. We have analyzed nucleotide composition, codon usage, compositional biases, and constructing models of the secondary structure of tRNAs. Besides, we also discussed the phylogenetic relationships with other representative gastropods. This snail mt genome is the first model in the family Camaenidae, thus it can offer worthwhile information to other camaenids.

## Materials and methods

### Genomic DNA extraction, PCR amplification and DNA sequencing

Adults of *Camaena cicatricosa* were collected from Xishan Park in Guiping (23°23'58"N, 110°3'46"E), Guangxi, China in November 2, 2013. Specimens were initially preserved in 100% ethanol in the field, and then stored at -20 °C at Fujian Entry-Exit Inspection & Quarantine Bureau (FJCIQ). Total genomic DNA was extracted from the pedal muscle tissue of single individual using the DNeasy Blood and Tissue kit (Qiagen) according to the manufacturer’s instructions. Voucher specimen (FJCIQ 18483) is deposited at the Key Laboratory of Molluscan Quarantine and Identification of AQSIQ, Fujian Entry-Exit Inspection & Quarantine Bureau, Fuzhou, Fujian.

The entire genome was successfully amplified by polymerase chain reaction (PCR) in overlapping fragments with four pairs of mitochondrial universal primers from previous works (Palumbi et al. 1991; Folmer et al. 1994; Merritt et al. 1998; Hugall et al. 2002), and four pairs of perfectly matched specific primers designed from sequenced short fragments in this study (Table 1). Short PCRs (< 2 kb) were performed using Takara Taq DNA polymerase (TaKaRa, Dalian, China), with the following cycling conditions: 30 s at 94 °C, followed by 35 cycles of 10 s at 94 °C, 50 s at 40 °C or 45 °C, and 1 min at 72 °C. The final elongation step was continued for 10 min at 72 °C. Long range PCRs (> 4 kb) were performed using Takara Long Taq DNA polymerase (TaKaRa, Dalian, China) under the following cycling conditions: 1 min at 94 °C, followed by 40 cycles of 10s at 98 °C, 50 s at 60 °C, 4−8 min at 68 °C, and the final elongation step at 72 °C for 6 min. The PCR products were checked by spectrophotometry and 1.0% agarose gel electrophoresis.

**Table 1.** Primer pairs used for PCR amplification.

No. of fragment	Primer name	Nucleotide sequence (5’–3’) and location	Size (bp)	Reference
1	LCO-1490	GGTCAACAAATCATAAAGATATTGG		Folmer et al. 1994
	HCO-2198	TAAACTTCAGGGTGACCAAAAAATCA		Folmer et al. 1994
2	Fcoi	TGAACTGTTTATCCTCCAC (364–382)	1908	Present study
	RL	TAGGGTCTTCTCGTCTTT (2254–2271)		Present study
3	16Sar-L	CGCCTGTTTATCAAAAACAT		Palumbi et al. 1991
	16Sbr-H	CCGGTCTGAACTCAGATCACGT		Palumbi et al. 1991
4	FL2	CGATGTTGGATTAGGAAGTTGA (2415–2436)	4267	Present study
	Rcb2	TAAAGGATTTGTTGACCCACG (6661–6681)		Present study
5	144F	TGAGSNCARATGTCNTWYTG		Merritt et al. 1998
	272R	GCRAANAGRAARTACCAYTC		Merritt et al. 1998
6	Fcb	GTGGGTCAACAAATCCTT (6662–6679)	816	Present study
	Rcoii	ATGAACACCTCGGGTAGT (7460–7477)		Present study
7	FCOII	AAATAATGCTATTTCATGAYCAYG		Hugall et al. 2002
	RCOII	GCTCCGCAAATCTCTGARCAYTG		Hugall et al. 2002
8	SF1F	AAATTCCATTAGAGGGGCTTATACGCCGCC (6984–7013)	6957	Present study
	SF1R	CAAGAGATAGTCCCGTACCAACTATGCCGC (68–79)		Present study

Short fragments were sequenced from both directions after purification using the BigDye Terminator Sequencing Kit (Applied Biosystems, San Francisco, CA, USA) and the ABI PRIMER^Tm^3730XL DNA Analyzer (PE Applied Biosystems) with internal primers for primer walking. For the long fragments, the shotgun libraries of *Camaena cicatricosa* were constructed, and then the positive clones were sequenced using above kit and sequenator with vector-specific primers *Bca*Best primer M13-47 and *Bca*Best Primer RV-M.

### Genome annotation and inference of secondary structure

Raw sequences were proof-read and aligned into contigs with BioEdit v.7.0.5.3 (Hall 1999). The tRNA genes were identified with tRNAscan-SE Search Server v.1.21 (Lowe and Eddy 1997) and DOGMA (Wyman et al. 2004), while others that could not be determined by these two tools were predicted by similarity comparison with other published land snails (Terrett et al. 1995; Yamazaki et al. 1997; Groenenberg et al. 2012; Gaitán-Espitia et al. 2013; He et al. 2014; Deng et al. 2014). The PCGs and rRNA genes were annotated by BLAST in Genbank with published available mitochondrial sequences of terrestrial snails.

PCGs were aligned with Clustal X (Thompson et al. 1997). The nucleotide composition and codon usage were analyzed with MEGA 5.0 (Tamura et al. 2011). Strand asymmetry was denoted by skew values, which were calculated according to the formulas: AT skew = [A−T]/[A+T] and GC skew = [G−C]/[G+C] (Perna and Kocher 1995).

Phylogenetic analyses were performed based on 11 representative gastropod mt genomes from GenBank (Table 2) using maximum likelihood (ML) and maximum parsimony (MP) methods. One species of Opisthobranchia was selected as outgroup. A DNA alignment with 9,892bp length was inferred from the amino acid alignment of 13 PCGs using MEGA 5.0 (Tamura et al. 2011). The selection of best-fit-substitution model for ML estimation was performed using MEGA 5.0 with corrected Akaike information criterion (AIC). Node supports for ML and MP analyses were calculated through 1000 bootstrap replicates. All other settings were kept as default.

**Table 2.** Summary of samples information used in this study.

Subclass /order	Family	Species	Accession number	Reference
Stylommatophora				
	Camaenidae	*Camaena cicatricosa*	KM365408	Present study
	Bradybaenidae	*Euhadra herklotsi*	Z71693–Z71701	Yamazaki et al. 1997
		*Mastigeulota kiangsinensis*	KM083123	Deng et al. 2014
	Helicidae	*Cornu aspersum*	JQ417195	Gaitán-Espitia et al. 2013
		*Cepaea nemoralis*	CMU23045	Terrett et al. 1995
		*Cylindrus obtusus*	JN107636	Groenenberg et al. 2012
	Succineidae	*Succinea putris*	JN627206	White et al. 2011
	Clausiliidae	*Albinaria caerulea*	X83390	White et al. 2011
	Achatinidae	*Achatina fulica*	NC024601	He et al. 2014
Basommatophora	Lymnaeidae	*Galba pervia*	JN564796	Liu et al. 2012
Opisthobranchia	Aplysiidae	*Aplysia californica*	AY569552	Knudsen et al. 2006

## Results and discussion

**Figure 1.**
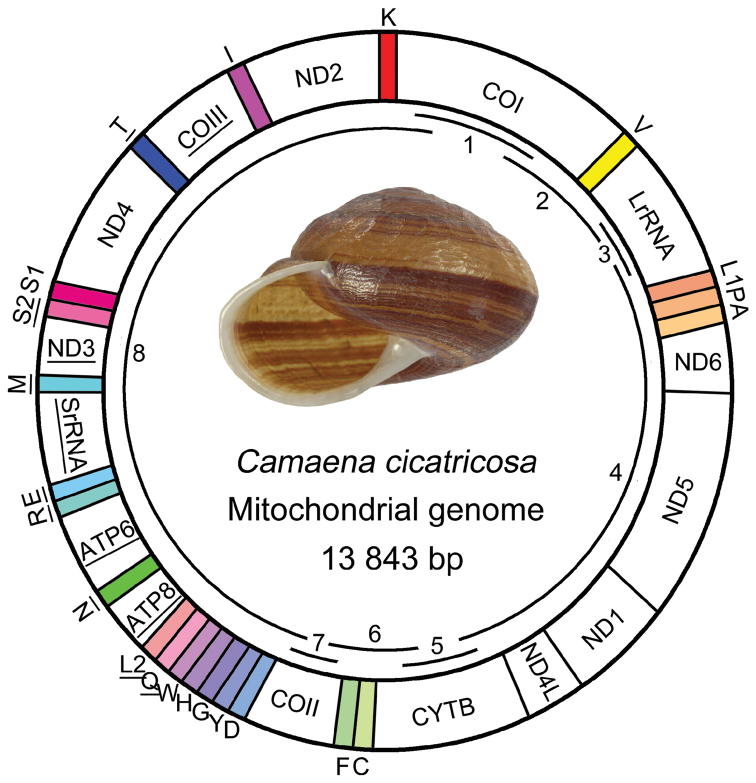
The mt genome of *Camaena cicatricosa*. The tRNA genes are labeled based on the IUPACIUB single letter amino acid codes. Genes with underline illuminate the direction of transcription from 3’ to 5’, and without underline illuminating from 5’ to 3’. Numbers and overlapping lines within the circle indicate PCR fragments amplified for sequencing (see Table 1).

**Figure 2.**
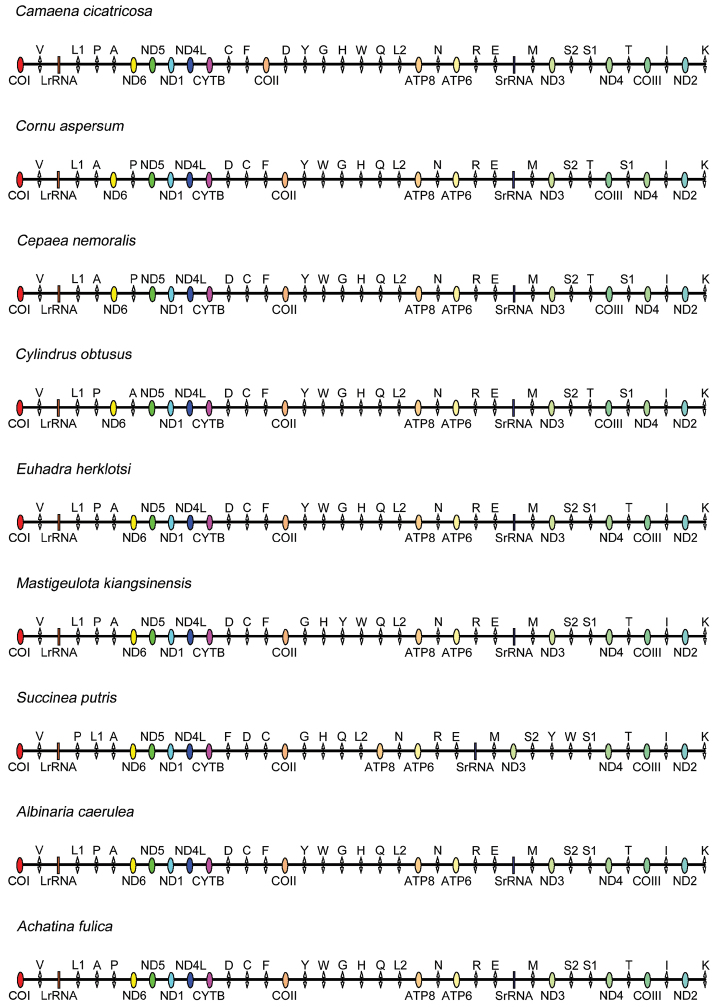
Gene arrangement of nine mt genomes in the order Stylommatophora.

The complete mt genome of *Camaena cicatricosa* was a double-stranded circular molecule of 13,843 bp in length (GenBank: KM365408). It contained 13 PCGs, 22 tRNA genes, two rRNA genes, similar to other mt genomes of land snails from the order Stylommatophora. All genes were divided into two groups, encompassing 24 genes on the majority coding strand (J strand) and others on the minority coding strand (N strand) (Fig. 1). However, the gene arrangement differed from that of the known land snails in the order Stylommatophora, specially the locations of *tRNA^Cys^*, *tRNA^Phe^*, *COII*, *tRNA^Asp^*, *tRNA^Gly^*, *tRNA^His^* and *tRNA^Trp^* (Fig. 2). Gene overlaps with a total of 242 bp were found at 16 gene junctions, and the longest overlap (50 bp) existed between *ND6* and *ND5*. Besides, there were 144 nucleotides dispersed in 14 intergenic spacers with the shortest 1 bp and the longest 29 bp. The 29 bp long noncoding region was situated between *COIII* and *tRNA^Ile^*; the shortest 1bp in three gene spacers (Table 3).

**Table 3.** Organization of the *Camaena cicatricosa* mt genome.

Gene	Direction	Location	Size (bp)	Anticodon	Start codon	Stop codon	Intergenic nucleotides
*COI*	F	1–1527	1527		ATG	TAG	
*tRNA^Val^*	F	1527–1585	59	1557–1559 TAC			–1
*lrRNA*	F	1586–2582	997				0
*tRNA^Leu(CUN)^*	F	2583–2642	60	2611–2613 TAG			0
*tRNA^Pro^*	F	2640–2702	63	2669–2671 TGG			–3
*tRNA^Ala^*	F	2705–2764	60	2735–2737 TGC			2
*ND6*	F	2784–3251	468		ATA	TAA	19
*ND5*	F	3202–4893	1692		ATT	TAA	–50
*ND1*	F	4914–5786	873		ATA	TAA	20
*ND4L*	F	5797–6072	276		ATA	TAA	10
*CytB*	F	6076–7188	1113		ATG	TAA	3
*tRNA^Cys^*	F	7185–7246	62	7215–7217 GCA			–4
*tRNA^Phe^*	F	7249–7309	61	7279–7281 GAA			2
*COII*	F	7310–7984	675		GTG	TAG	0
*tRNA^Asp^*	F	7989–8048	60	8019–8021 GTC			4
*tRNA^Tyr^*	F	8075–8136	62	8105–8107 GTA			26
*tRNA^Gly^*	F	8132–8191	60	8162–8164 TCC			–5
*tRNA^His^*	F	8188–8246	59	8218–8220 GTG			–4
*tRNA^Trp^*	F	8255–8314	60	8282–8284 TCA			8
*tRNA^Gln^*	R	8311–8369	59	8338–8340 TTG			–4
*tRNA^Leu(UUR)^*	R	8366–8429	64	8398–8400 TAA			–4
*ATP8*	R	8431–8595	165		ATG	TAA	1
*tRNA^Asn^*	R	8597–8652	56	8620–8622 GTT			1
*ATP6*	R	8652–9332	681		ATT	TAA	–1
*tRNA^Arg^*	R	9309–9366	58	9339–9341 TCG			–24
*tRNA^Glu^*	R	9366–9430	65	9393–9395 TTC			–1
*SrRNA*	R	9431–10112	682				0
*tRNA^Met^*	R	10113–10174	62	10140–10142 CAT			0
*ND3*	R	10165–10524	360		ATA	TAA	–10
*tRNA^Ser(UCN)^*	R	10517–10569	53	10548–10550 TGA			–8
*tRNA^Ser(AGN)^*	F	10570–10629	61	10594–10596 GCT			0
*ND4*	F	10648–11988	1341		ATA	TAA	18
*tRNA^Thr^*	R	11940–11999	60	11967–11969 TGT			–49
*COIII*	R	11965–12792	828		ATT	TAA	–35
*tRNA^Ile^*	F	12822–12885	64	12852–12854 GAT			29
*ND2*	F	12887–13828	942		ATG	TAA	1
*tRNA^Lys^*	F	13790–13843	54	13819–13821 TTT			–39

Note: Negative numbers indicate adjacent gene overlap.

### Protein coding genes

The length of PCGs was 10,941bp, accounting for 79.04% of the whole mt genome (Table 4). Most PCGs started with ATN as initiation codons (four with ATG, three with ATT, and five with ATA) except for *COII* gene with GTG (Table 3), while ATC, TTA, TTG, CTT and TCG as unconventional start signals have been found in other invertebrates (Raay and Crease 1994; Crease 1999; Yamazaki et al. 1997; Yu et al. 2007; Groenenberg et al. 2012). Conventional stop codons TAA and TAG had been assigned to all PCGs (Table 3). However, an incomplete terminator signal (T) has been found in other snails (Terrett et al. 1995; Hatzoglou et al. 1995; Yamazaki et al. 1997; White et al. 2011; Groenenberg et al. 2012; Gaitán-Espitia et al. 2013).

**Table 4.** Nucleotide composition and skew of the *Camaena cicatricosa* mt genome.

	Proportion of nucleotides							
Feature	%A	%T	%G	%C	%A+T	AT Skew	GC Skew	No. of nucleotides
Whole genome	31.90	37.90	16.70	13.50	69.80	–0.09	0.11	13843
Protein coding genes	31.18	38.14	17.05	13.64	69.32	–0.10	0.11	10941
Protein coding genes (J)	28.83	40.41	17.54	13.23	69.24	–0.17	0.14	8907
Protein coding genes (N)	28.22	41.45	15.44	14.90	69.67	–0.19	0.02	2034
tRNA genes	34.95	36.46	15.81	12.78	71.41	–0.02	0.11	1322
tRNA genes (J)	33.96	36.80	17.51	11.72	70.77	–0.04	0.20	845
tRNA genes (N)	35.85	36.69	14.68	12.79	72.54	–0.01	0.07	477
rRNA genes	35.14	37.28	14.83	12.75	72.42	–0.03	0.08	1679

### Transfer RNA genes

**Figure 3.**
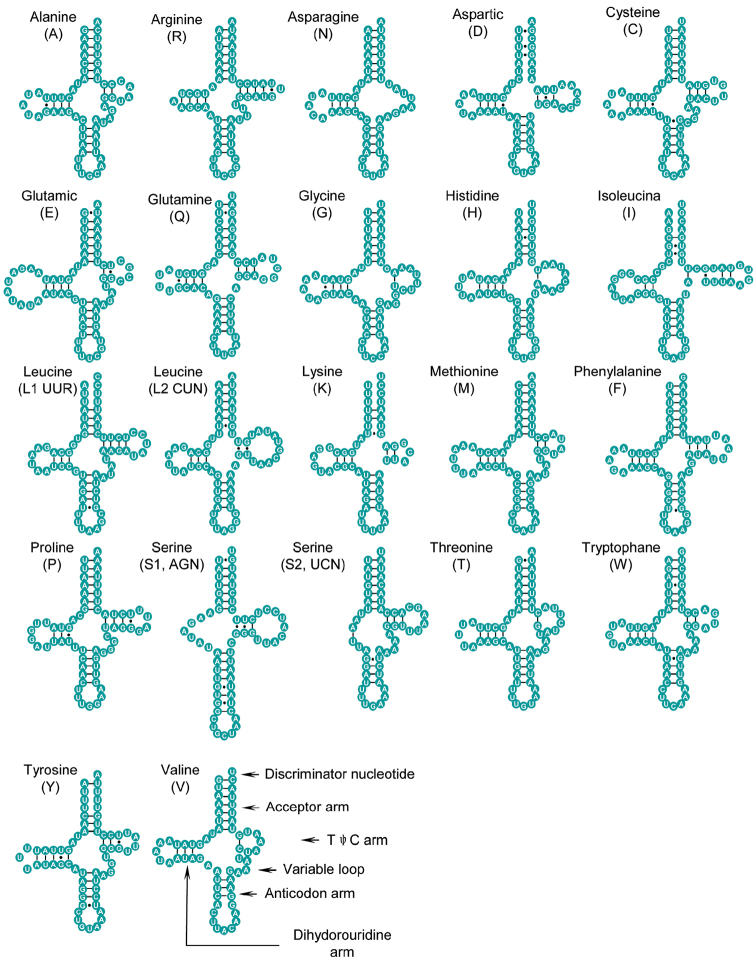
Inferred secondary structures of 22 tRNA genes in *Camaena cicatricosa*. Dashed (-) indicates Watson-Crick base pairing and (•) indicates G-U base pairing.

The 22 tRNA genes typically found in metazoan mt genomes were also discovered in *Camaena cicatricosa*, and 18 of them were determined by tRNAscan-SE (Lowe and Eddy 1997) and DOGMA (Wyman et al. 2004). The other four tRNA genes that could not be detected by the two programs were identified and drawn through comparison with known patterns of previous researches (Terrett et al. 1995; Grande et al. 2002; Groenenberg et al. 2012; Gaitán-Espitia et al. 2013). Fourteen tRNA genes were encoded on the J strand and the remainings on the N strand. Most tRNA genes could be folded into classic clover leaf structures exclusive of *tRNA^Asn^* and *tRNA^Ser(AGN)^*, in which their TψC arm and dihydrouridine (DHU) arm simply formed a loop (Fig. 3).

The length of tRNA genes ranged from 53 to 65 bp (Table 3). All amino acid acceptor (AA) arms (7 bp), anticodon (AC) loops (7 bp) and arms (5 bp) were almost invariant. However, other arms and loops changed considerably in size. Additionally, in some tRNA genes, non-Watson-Crick matches and aberrant loops had been found. For example, a total of 73 unmatched base pairs existed in some tRNAs, and 38 of them were G-U pairs, situated in the AA stem (13 bp), the T stem (10 bp), the AA stem (8 bp) and the DHU stem (7 bp). The remaining five base pairs included U-U mismatches, U-C mismatches, A-C mismatches, A-G mismatches and A-A mismatches (Fig. 3). Nevertheless, the post-transcriptional RNA-editing mechanism can rectify these mismatches to maintain tRNA functions (Tomita et al. 2001).

### Ribosomal RNA genes

The rRNA genes comprising large rRNA subunit (*lrRNA*) and small rRNA subunit (*srRNA*) are presumed to block in the spaces of flanking genes (Boore 2001; 2006). The *lrRNA* gene was situated between *tRNA^Val^* and *tRNA^Leu(CUN)^* revealing 78.23% consistency with *Euhadra herklotsi* and *Mastigeulota kiangsinensis*. The *srRNA* gene was located between *tRNA^Glu^* and *tRNA^Met^* (Fig. 1). The length of them were determined to be 997 bp and 682 bp respectively (Table 3).

### Base composition and codon usage

**Figure 4.**
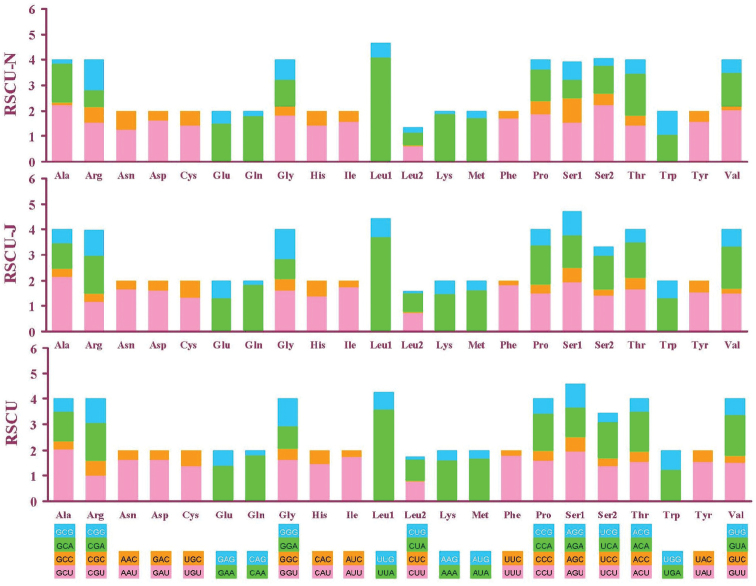
Relative synonymous codon usage (RSCU) in the *Camaena cicatricosa* mt genome. Codon families are provided on the x axis.

Like other snail mt genomes, the nucleotide composition of the *Camaena cicatricosa* mt genome was obviously biased toward adenine and thymine (A = 31.90%, T = 37.90%, C = 13.50%, G = 16.70%). The entire mt genome had a high A+T content of 69.80%, by the composition of 69.32% in PCGs, 71.41% in tRNA genes, 72.42% in rRNA genes. Nucleotide bias can also be reflected by codon usage. Evidently, we can see that NNA and NNU were applied frequently in most PCGs. Furthermore, codons TTT (phenylalanine), TTA (leucine), ATT (isoleucine) and ATA (methionine) which were used widely were all composed of A and T. Especially, more and more codons were biased in favor of those codons with A or T in the third position (Fig. 4).

The nucleotide composition of metazoan mt genomes usually demonstrate an obvious strand bias (Hassanin et al. 2005; Hassanin 2006) that can be described as AT and GC skews (Perna and Kocher 1995). The PCGs skew statistics of *Camaena cicatricosa* showed a great TA skew and nearly equal G and C on the N strand, whereas a great GC skew on the J strand. The nucleotide composition of tRNAs on the J strand were GC and TA skews, evidently exceeding values on the N strand (Table 4). AT and GC skews of *Camaena cicatricosa* mt genome differ from the strand biases of metazoan mtDNA (generally positive AT skew and negative GC skew for the J strand, contrary to the N strand for most metazons).

### Noncoding regions

The noncoding regions of *Camaena cicatricosa* mt genome contained some short intergenic spacers. These short sequences possibly acted as splicing recognition sites during the process of transcription (He et al. 2005). In the sequenced complete mt genome of the order Stylommatophora, the short intergenic spacers range from 1 bp to 65 bp (Hatzoglou et al. 1995; Terrett et al. 1995; Yamazaki et al. 1997; White et al. 2011; Groenenberg et al. 2012; Gaitán-Espitia et al. 2013; Deng et al. 2014) except *Achatina fulica* with 551 bp long noncoding region (He et al. 2014). However, the longest noncoding region was only 29 bp in *Camaena cicatricosa*. The shorter lengths of noncoding regions indicated that the mt genome of stylommatophorans are quit compact.

A large noncoding region called control region or AT-rich region is commonly seen in metazoan mt genomes (Boore 1999). In fact, variation of size for the entire mt genome can be chalked up to the presence of a number of tandem repeats (Zhang and Hewitt 1997) in control region, which may be caused by replication slippage (Levinson and Gutman 1987; Fumagalli et al. 1996). Nevertheless, putative control region (POR) was not aligned confidently in gastropods (Groenenberg et al. 2012) except *Achatina fulica* having a 551 bp POR between *COI* and *tRNA^Val^* (He et al. 2014). Other eight stylommatophoran species may possess short POR regions located adjacent to *COIII* (Hatzoglou et al. 1995; Terrett et al. 1995; Yamazaki et al. 1997; White et al. 2011; Groenenberg et al. 2012; Gaitán-Espitia et al. 2013; Deng et al. 2014). The POR regions of three helicid species and *Mastigeulota kiangsinensis* were located between *COIII* and *tRNA^Ser^* with lengths of 158–189 bp, whereas in the other three species were located between *COIII* and *tRNA^Ile^* with lengths of 42–47 bp. The 29 bp noncoding region of *Camaena cicatricosa* was located between *COIII* and *tRNA^Ile^*, but its length was shorter than other stylommatophorans.

### Phylogenetic analysis

**Figure 5.**
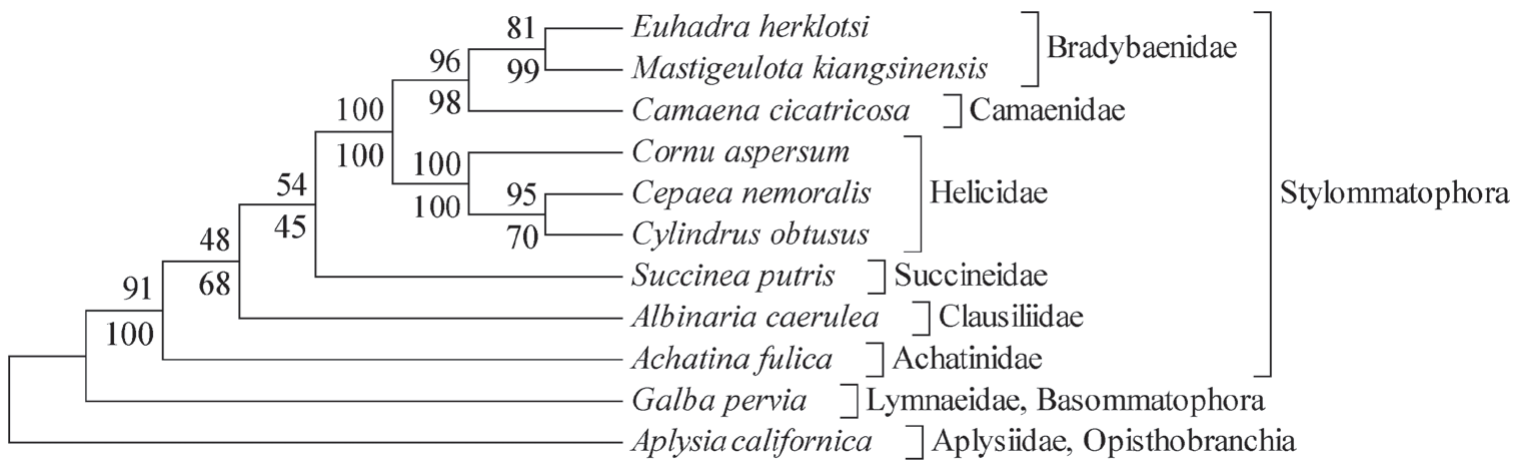
Phylogenetic tree inferred by maximum likelihood (ML) and maximum parsimony (MP) methods based on 13 protein genes. The tree is rooted with *Aplysia californica*. Numbers on or under the nodes represent bootstrap values of MP and ML respectively.

ML tree was estimated according to the GTR+G+I substitution model selected by AIC. The ML and MP trees (Fig. 5) displayed the same topologies and presented eight major clades corresponding to the families Bradybaenidae, Camaenidae, Helicidae, Succineidae, Clausiliidae, Achatinidae, Lymnaeidae and Aplysiidae. The monophyly of Stylommatophora was approved. Species in Helicidae were sister groups and congruent with previous works (Gaitán-Espitia et al. 2013). *Camaena cicatricosa* and *Mastigeulota kiangsinensis* from China and *Euhadra herklotsi* from Japan are monophyletic. However, the systematics of the families Camaenidae, Helicidae and Bradybaenidae are complicated and not fully resolved. Systematic and phylogenetic studies based on analyses of morphological versus molecular markers have produced inconsistent results (Scott 1996; Cuezzo 2003; Wade et al. 2007; Hirano et al. 2014). A final assessment of the systematic relationships of the three families is pending requiring a more complete taxon sampling.
